# Supplementary material for: Nanobodies against C. difficile TcdA and TcdB reveal unexpected neutralizing epitopes and provide a toolkit for toxin quantitation in vivo
Source: PLoS Pathog. 2023 Oct 23;19(10):e1011496. doi: 10.1371/journal.ppat.1011496 (PMC10621975; doi:10.1371/journal.ppat.1011496)
Supplement: S1 Fig — Highly identical clones from each panel were removed (TcdA cutoff 100% ID, TcdB cutoff 95% ID) for analysis. Total clone numbers for each group are in parentheses. Nanobodies used in the experiments in this study are highlighted in yellow. Domain specificity was determined by ELISA (red circle GTD, blue square DD or APD-DD, green star CROPs). (DOCX) [file ppat.1011496.s001.docx]

**B.**

**A.**

**S1 Fig. Cladograms representing amino acid sequence analysis and domain specificity of TcdA (*A*) and TcdB (*B*) nanobodies.** Highly identical clones from each panel were removed (TcdA cutoff 100% ID, TcdB cutoff 95% ID) for analysis. Total clone numbers for each group are in parentheses. Nanobodies used in the experiments in this study are highlighted in *yellow*. Domain specificity was determined by ELISA (*red circle* GTD, *blue square* DD or APD-DD, *green star* CROPs).
