# Supplementary material for: Nanobodies against C. difficile TcdA and TcdB reveal unexpected neutralizing epitopes and provide a toolkit for toxin quantitation in vivo
Source: PLoS Pathog. 2023 Oct 23;19(10):e1011496. doi: 10.1371/journal.ppat.1011496 (PMC10621975; doi:10.1371/journal.ppat.1011496)
Supplement: S2 Fig — Analysis was performed in GraphPad Prism by least squares fit of the model: log(agonist) vs. response—variable slope (four parameters). Error bars represent standard error for triplicate experiments, where possible. (DOCX) [file ppat.1011496.s002.docx]

**S2 Fig. Data graphs of toxin neutralization assays for TcdA (top) or TcdB (bottom).** Analysis was performed in GraphPad Prism by least squares fit of the model: log(agonist) vs. response -- variable slope (four parameters). Error bars represent standard error for triplicate experiments, where possible.
