# Supplementary material for: Nanobodies against C. difficile TcdA and TcdB reveal unexpected neutralizing epitopes and provide a toolkit for toxin quantitation in vivo
Source: PLoS Pathog. 2023 Oct 23;19(10):e1011496. doi: 10.1371/journal.ppat.1011496 (PMC10621975; doi:10.1371/journal.ppat.1011496)
Supplement: S3 Fig — Detection of purified, recombinant TcdA by sandwich ELISA using: A) capture Nb A1C3 (anti-DD) and detection Nb A2B10 (anti-CROPs), B) capture Nb A2B10 (anti-CROPs) and detection Nb A1C3 (anti-DD), C) capture Nb A1D1 (anti-DD) and detection Nb A1C3 (anti-DD), and D) capture Nb A2B5 (anti-DD) and detection Nb A1C3 (anti-DD). Two-fold serial dilutions of rTcdA were used, except where noted. All ELISAs were performed in biological triplicate and error bars represent standard error of the mean (SEM). Image created with Biorender.com license number PU25IG5KBX. (DOCX) [file ppat.1011496.s003.docx]

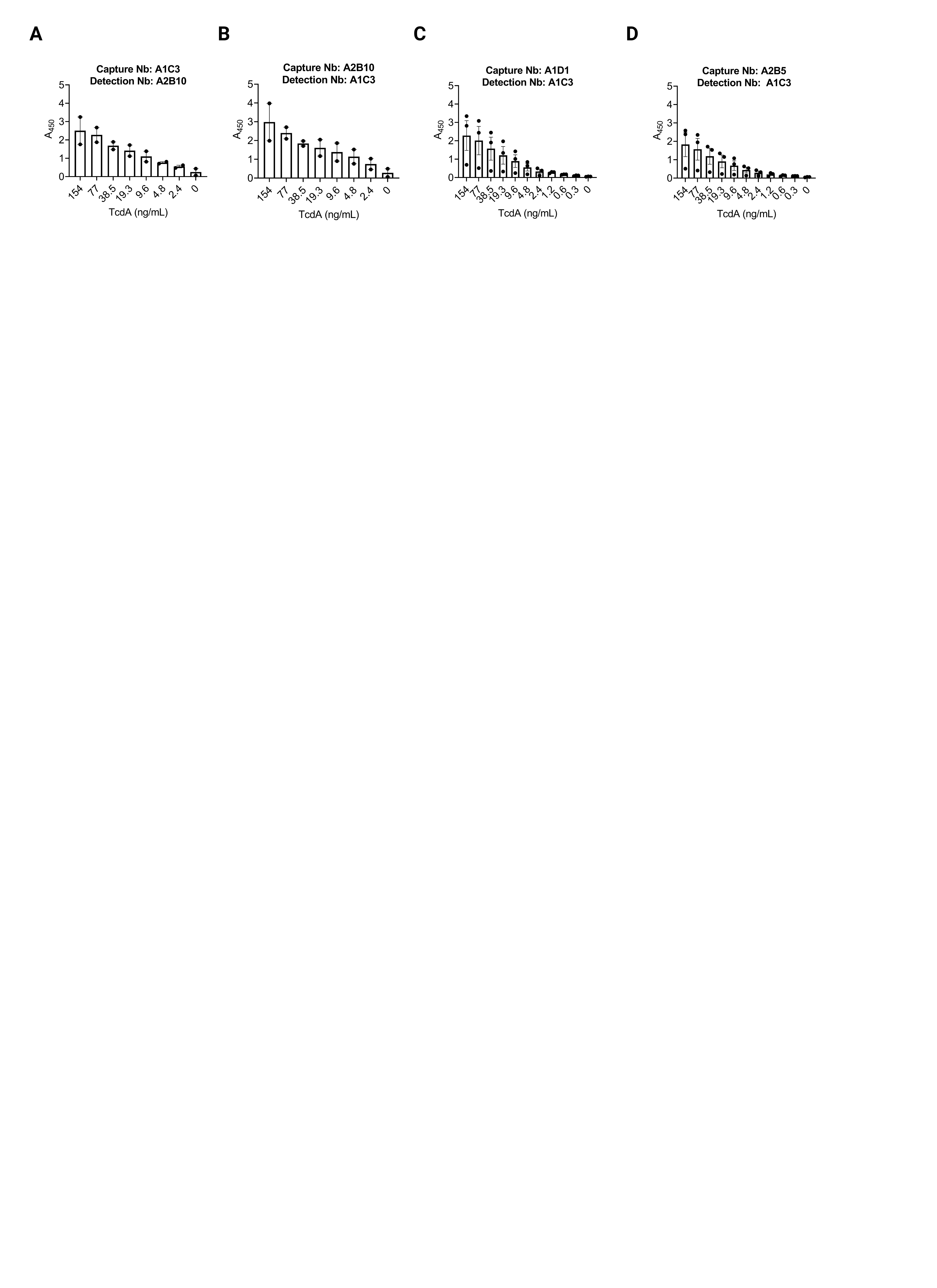


**S3 Fig. Screening nanobody pairs for anti-TcdA ELISAs.** Detection of purified, recombinant TcdA by sandwich ELISA using: A) capture Nb A1C3 (anti-DD) and detection Nb A2B10 (anti-CROPs), B) capture Nb A2B10 (anti-CROPs) and detection Nb A1C3 (anti-DD), C) capture Nb A1D1 (anti-DD) and detection Nb A1C3 (anti-DD), and D) capture Nb A2B5 (anti-DD) and detection Nb A1C3 (anti-DD). Two-fold serial dilutions of rTcdA were used, except where noted. All ELISAs were performed in biological triplicate and error bars represent standard error of the mean (SEM). Image created with Biorender.com license number PU25IG5KBX*.*
