# Supplementary material for: Nanobodies against C. difficile TcdA and TcdB reveal unexpected neutralizing epitopes and provide a toolkit for toxin quantitation in vivo
Source: PLoS Pathog. 2023 Oct 23;19(10):e1011496. doi: 10.1371/journal.ppat.1011496 (PMC10621975; doi:10.1371/journal.ppat.1011496)
Supplement: S4 Fig — Mice were infected with 104 spores of R20291 TcdBGTX, sacrificed after 2 days, weighed, and cecal contents and fecal contents were collected for TcdA/TcdB quantification. A, B) Individual mouse fecal or D, E) cecal toxin quantities from Figs 3 and 4 were plotted against weight loss (expressed as a percent of starting weight). In addition, individual C) fecal or F) cecal TcdA quantities were plotted against the corresponding TcdB quantities. Image created with Biorender.com license number AK25IG337P. (DOCX) [file ppat.1011496.s004.docx]

**
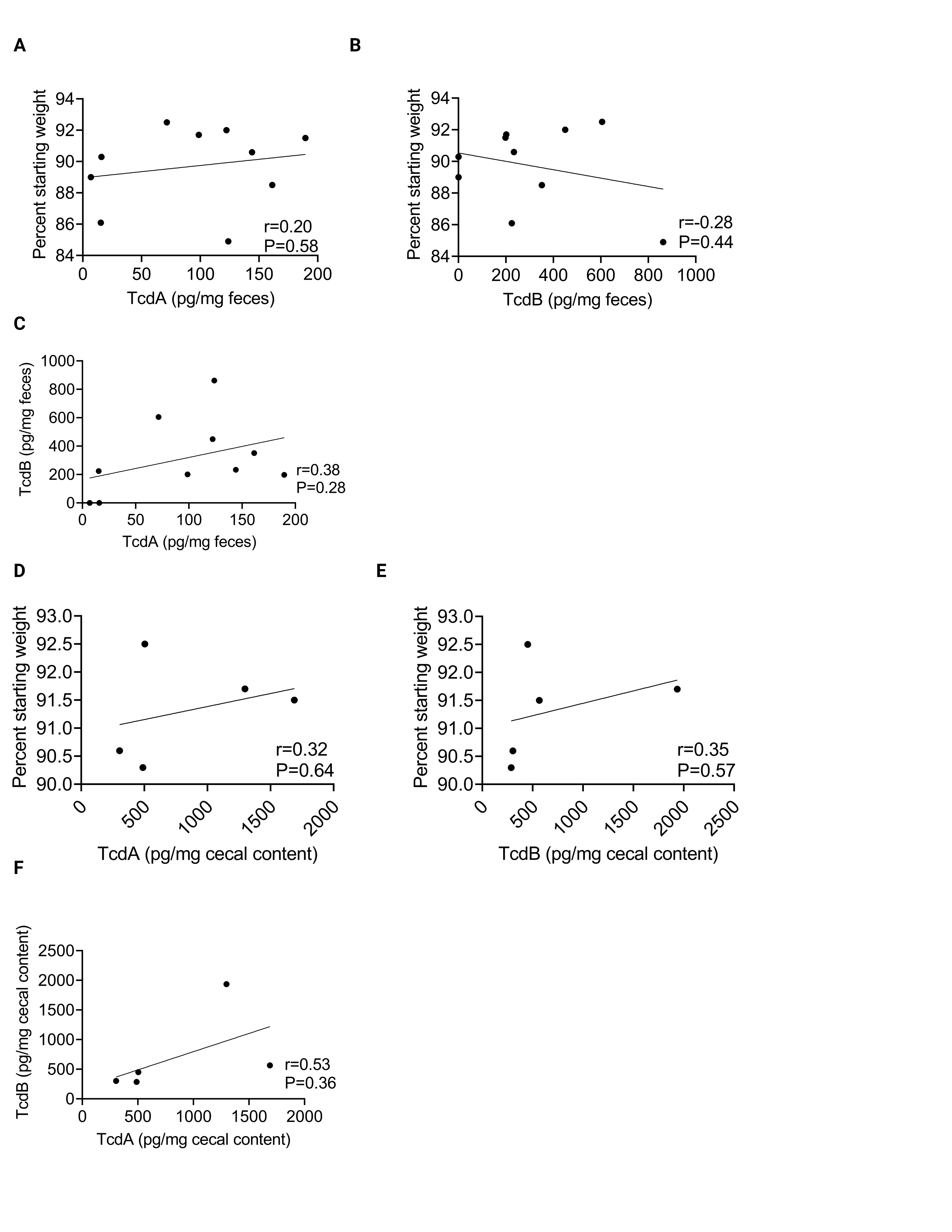
**

**S4 Fig. Comparison of toxin concentrations and weight loss in a murine model of *C. difficile* infection.** Mice were infected with 10^4^ spores of R20291 TcdB_GTX_, sacrificed after 2 days, weighed, and cecal contents and fecal contents were collected for TcdA/TcdB quantification. A, B) Individual mouse fecal or D, E) cecal toxin quantities from Figs 3 and 4 were plotted against weight loss (expressed as a percent of starting weight). In addition, individual C) fecal or F) cecal TcdA quantities were plotted against the corresponding TcdB quantities. Image created with Biorender.com license number AK25IG337P*.*
