# Supplementary material for: Nanobodies against C. difficile TcdA and TcdB reveal unexpected neutralizing epitopes and provide a toolkit for toxin quantitation in vivo
Source: PLoS Pathog. 2023 Oct 23;19(10):e1011496. doi: 10.1371/journal.ppat.1011496 (PMC10621975; doi:10.1371/journal.ppat.1011496)
Supplement: S5 Fig — Detection of purified, recombinant TcdB (1 nM) from C. difficile VPI10463 (a TcdB1 strain) or R20291 (a TcdB2 strain), labeled rTcdBVPI or rTcdB027, by sandwich ELISA using: A) capture Nb B2C11 (anti-GTD) and detection Nb B1A11 (anti-CROPs), B) capture Nb B2F11 (anti-CROPs) and detection Nb B0E2 (anti-DD), C) capture Nb B2F11 (anti-CROPs) and detection Nb B1A11 (anti-CROPs), and D) capture Nb B2F11 (anti-CROPs) and detection Nb B0B11 (anti-GTD). Evaluation of anti-DD Nbs E) B0A12/B0E2 and F) B1C11/B0E2 in the sandwich ELISA assay using capture Nb B0A12 or B1C11, respectively, and detection Nb B0E2. B0A12 or B1C11 was used to coat the plate, followed by two-fold serial dilutions of rTcdBVPI or rTcdB027 except where noted. All ELISAs were performed in biological triplicate and error bars represent standard error of the mean (SEM). Image created with Biorender.com license number CQ25IG5GXK. (DOCX) [file ppat.1011496.s005.docx]

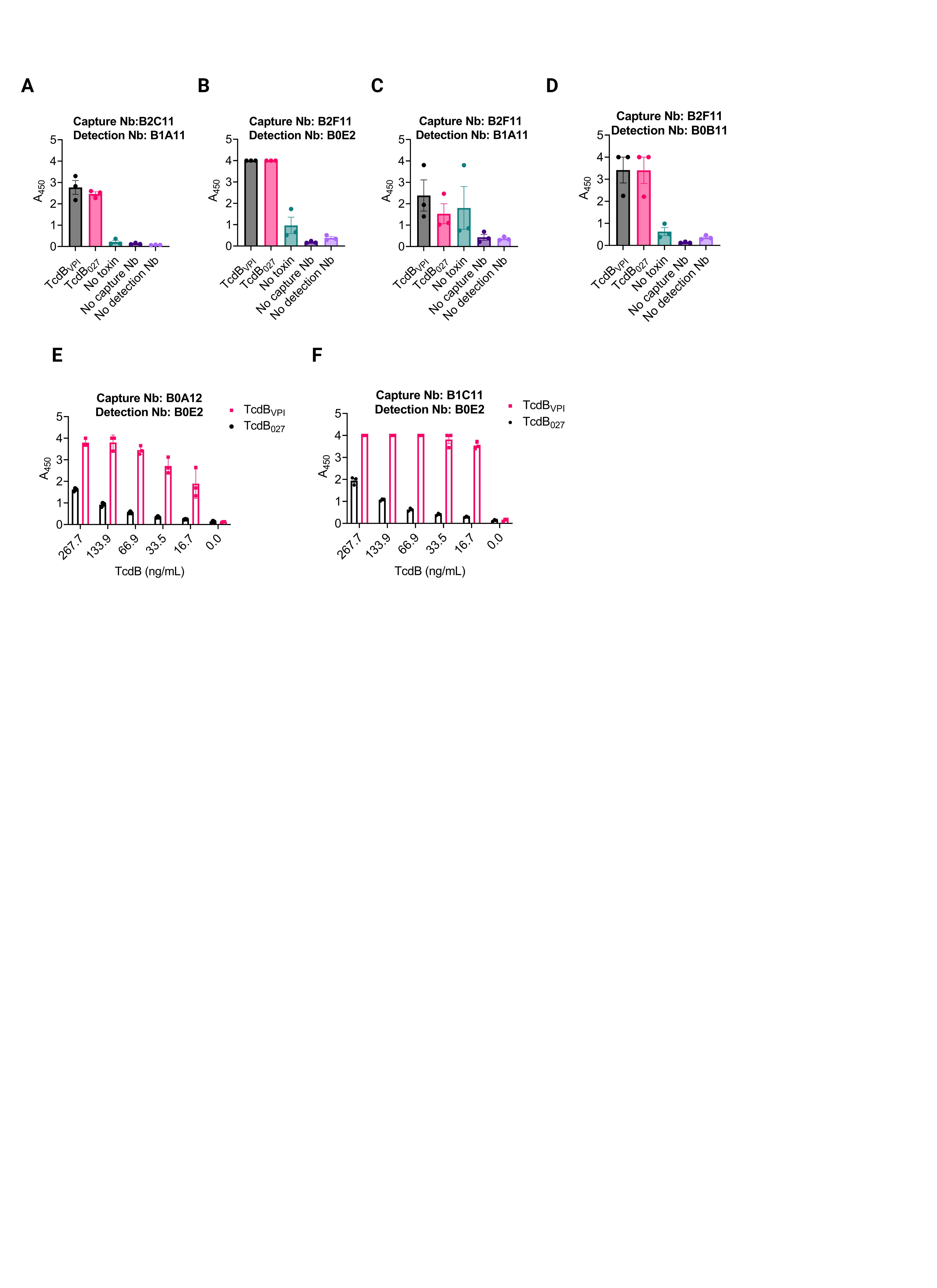


**S5 Fig. Screening nanobody pairs for anti-TcdB ELISAs.** Detection of purified, recombinant TcdB (1 nM) from *C. difficile* VPI10463 (a TcdB1 strain) or R20291 (a TcdB2 strain), labeled rTcdB_VPI_ or rTcdB_027_, by sandwich ELISA using: A) capture Nb B2C11 (anti-GTD) and detection Nb B1A11 (anti-CROPs), B) capture Nb B2F11 (anti-CROPs) and detection Nb B0E2 (anti-DD), C) capture Nb B2F11 (anti-CROPs) and detection Nb B1A11 (anti-CROPs), and D) capture Nb B2F11 (anti-CROPs) and detection Nb B0B11 (anti-GTD). Evaluation of anti-DD Nbs E) B0A12/B0E2 and F) B1C11/B0E2 in the sandwich ELISA assay using capture Nb B0A12 or B1C11, respectively, and detection Nb B0E2. B0A12 or B1C11 was used to coat the plate, followed by two-fold serial dilutions of rTcdB_VPI_ or rTcdB_027_ except where noted. All ELISAs were performed in biological triplicate and error bars represent standard error of the mean (SEM). Image created with Biorender.com license number CQ25IG5GXK.
