# Supplementary material for: Nanobodies against C. difficile TcdA and TcdB reveal unexpected neutralizing epitopes and provide a toolkit for toxin quantitation in vivo
Source: PLoS Pathog. 2023 Oct 23;19(10):e1011496. doi: 10.1371/journal.ppat.1011496 (PMC10621975; doi:10.1371/journal.ppat.1011496)
Supplement: S1 Table — (DOCX) [file ppat.1011496.s006.docx]

S1 Table. Nanobody Sequences

Nanobody sequences with complementary determining regions colored red.
