# Supplementary material for: Nanobodies against C. difficile TcdA and TcdB reveal unexpected neutralizing epitopes and provide a toolkit for toxin quantitation in vivo
Source: PLoS Pathog. 2023 Oct 23;19(10):e1011496. doi: 10.1371/journal.ppat.1011496 (PMC10621975; doi:10.1371/journal.ppat.1011496)
Supplement: S2 Table — (DOCX) [file ppat.1011496.s007.docx]

| **S2 Table. Plasmids used in this study** | | | |
| --- | --- | --- | --- |
| **Number** | **Plasmid** | **Relevant characteristics** | **Source** |
|  | ***Parent Plasmids*** |  |  |
|  | pC-His1622 | Low copy plasmid for protein overexpression in *Bacillus megaterium,* C-terminal 6X-histidine tag, inducible *P_xylR_*, Tet^r^, Amp^r^ | Lab stock |
|  | pET28a/b/c(+) | Low copy plasmid for protein overexpression, N- and C-terminal 6X-histidine tag, inducible with *lacI*, Kan^r^ | Lab stock |
|  | pBG101 | Low copy plasmid for protein overexpression, pET27 derivative, N-terminal 6X-histidine tag, inducible with *lacI*, Kan^r^ | Lab stock |
|  | pBBR3 | Modified pADL22 plasmid, C-terminal HA-tag and 6X-histidine tag, inducible with *lacI*, Amp^r^ | Lab stock |
|  | ***Toxin Constructs*** |  |  |
|  | *TcdA* |  |  |
| pBL282 | pC-His1622-*tcdA* | pC-HIS1622, TcdA_1-2710_ | Lab stock |
| pBL764 | pC-His1622-*tcdA*_D285N/D287N_ | pC-HIS1622, TcdA_1-2710_ with mutations in the glucosyltransferase domain (D285N, D287N) | Lab stock |
| pBL515 | pC-His1622-*tcdA*_1-1832_ | pC-HIS1622, TcdA_1-1832_ (no CROPs domain) | Lab stock |
| pBL657 | pC-His1622-*tcdA*_1-1809_ | pC-HIS1622, TcdA_1-1809_ (no CROPs domain) | Lab stock |
| pBL500 | pC-His1622-*tcdA*_1-542_ | pC-HIS1622, TcdA_1-542_ (glucosyltransferase domain only) | Lab stock |
| pBL840 | pET28a(+)-*tcdA*_2460-2710_ | pET28a(+), TcdA_2460-2710_ (CROPs repeats 6-7) | Lab stock |
|  | *TcdB* |  |  |
| pBL377 | pC-His1622-*tcdB* | pC-HIS1622, TcdB_1-2366_ | Lab stock |
| pBL682 | pC-His1622-*tcdB*_L1106K_ | pC-HIS1622, TcdB_1-2366_ with point mutation L1106K | Lab stock |
| pBL832 | pC-His1622-*tcdB*_1-1810_ | pC-HIS1622, TcdB_1-1810_ (no CROPs domain) | Lab stock |
| pBL834 | pC-His1622-*tcdB*_1-543_ | pC-HIS1622, TcdB_1-543_ (glucosyltransferase domain only) | Lab stock |
| pBL757 | pET28b(+)-*tcdB*_842-1834_ | pET28b(+), TcdB_842-1834_ (delivery domain only) | Lab stock |
| pBL281 | pBG101-*tcdB*_1832-2366_ | pBG101, TcdB_1827-2366_ (CROPs domain only) | Lab stock |
|  | ***Nanobodies*** |  |  |
|  | *TcdA* |  |  |
| pNB126 | pBBR3-*A1A3* | pBBR3, nanobody from plate A1 location A3 | This study |
| pNB104 | pET28b(+)-*A1A6* | pET28b(+), nanobody from plate A1 location A6 | This study |
| pNB105 | pET28a(+)-*A1C1* | pET28a(+), nanobody from plate A1 location C1 | This study |
| pNB106 | pET28a(+)-*A1C3* | pET28a(+), nanobody from plate A1 location C3 | This study |
| pNB127 | pBBR3-*A1C4* | pBBR3, nanobody from plate A1 location C4 | This study |
| pNB128 | pBBR3-*A1C11* | pBBR3, nanobody from plate A1 location C11 | This study |
| pNB129 | pBBR3-*A1D1* | pBBR3, nanobody from plate A1 location D1 | This study |
| pNB130 | pBBR3-*A1D8* | pBBR3, nanobody from plate A1 location D8 | This study |
| pNB131 | pBBR3-*A1F4* | pBBR3, nanobody from plate A1 location F4 | This study |
| pNB132 | pBBR3-*A1G4* | pBBR3, nanobody from plate A1 location G4 | This study |
| pNB133 | pBBR3-*A1G6* | pBBR3, nanobody from plate A1 location G6 | This study |
| pNB107 | pET28a(+)-*A1H1* | pET28a(+), nanobody from plate A1 location H1 | This study |
| pNB134 | pBBR3-*A1H5* | pBBR3, nanobody from plate A1 location H5 | This study |
| pNB135 | pBBR3-*A2A6* | pBBR3, nanobody from plate A2 location A6 | This study |
| pNB109 | pET28a(+)-*A2A8* | pET28a(+), nanobody from plate A2 location A8 | This study |
| pNB110 | pET28a(+)-*A2B10* | pET28a(+), nanobody from plate A2 location B10 | This study |
| pNB136 | pBBR3-*A2B5* | pBBR3, nanobody from plate A2 location B5 | This study |
| pNB137 | pBBR3-*A2C2* | pBBR3, nanobody from plate A2 location C2 | This study |
| pNB111 | pET28a(+)-*A2F10* | pET28a(+), nanobody from plate A2 location F10 | This study |
| pNB138 | pBBR3-*A2F12* | pBBR3, nanobody from plate A2 location F12 | This study |
| pNB112 | pET28a(+)-*A2G1* | pET28a(+), nanobody from plate A2 location G1 | This study |
| pNB113 | pET28a(+)-A2G5 | pET28a(+), nanobody from plate A2 location G5 | This study |
| pNB139 | pBBR3-*A2G6* | pBBR3, nanobody from plate A2 location G6 | This study |
| pNB140 | pBBR3-*A2H4* | pBBR3, nanobody from plate A2 location H4 | This study |
| pNB141 | pBBR3-*A2H9* | pBBR3, nanobody from plate A2 location H9 | This study |
|  | *TcdB* |  |  |
| pNB007 | pBBR3*-B0A9* | pBBR3, nanobody from plate B0 location A9 | This study |
| pNB010 | pBBR3*-B0A12* | pBBR3, nanobody from plate B0 location A12 | This study |
| pNB017 | pBBR3-*B0B7* | pBBR3, nanobody from plate B0 location B7 | This study |
| pNB021 | pBBR3-*B0B11* | pBBR3, nanobody from plate B0 location B11 | This study |
| pNB032 | pBBR3*-B0C10* | pBBR3, nanobody from plate B0 location C10 | This study |
| pNB037 | pBBR3-*B0D3* | pBBR3, nanobody from plate B0 location D3 | This study |
| pNB044 | pBBR3-*B0D10* | pBBR3, nanobody from plate B0 location D10 | This study |
| pNB045 | pBBR3-*B0D11* | pBBR3, nanobody from plate B0 location D11 | This study |
| pNB048 | pBBR3-*B0E2* | pBBR3, nanobody from plate B0 location E2 | This study |
| pNB114 | pET28c(+)-*B1A11* | pET28c(+), nanobody from plate B1 location A11 | This study |
| pNB116 | pET28c(+)*-B1C10* | pET28c(+), nanobody from plate B1 location C10 | This study |
| pNB117 | pET28c(+)-*B1C11* | pET28c(+), nanobody from plate B1 location C11 | This study |
| pNB118 | pET28c(+)-*B1E7* | pET28c(+), nanobody from plate B1 location E7 | This study |
| pNB120 | pET28c(+)-*B2C5* | pET28c(+), nanobody from plate B2 location C5 | This study |
| pNB122 | pET28c(+)-*B2C11* | pET28c(+), nanobody from plate B2 location C11 | This study |
| pNB124 | pET28c(+)-*B2F11* | pET28c(+), nanobody from plate B2 location F11 | This study |
|  | *Avi-tagged* |  |  |
| pNB142 | pET28b(+)-*A1A6-Avi* | pET28b(+), nanobody from plate A1 location A6, C-terminal HA-tag, Avi-tag, and 5X histidine tag | This study |
| pNB143 | pET28b(+)-*A2B10-Avi* | pET28b(+), nanobody from plate A2 location B10, C-terminal HA-tag, Avi-tag, and 6X histidine tag | This study |
| pNB146 | pET28b(+)-*B0E2-Avi* | pET28b(+), nanobody from plate B0 location E2, C-terminal HA-tag, Avi-tag, and 6X histidine tag | This study |
