# Supplementary material for: Nanobodies against C. difficile TcdA and TcdB reveal unexpected neutralizing epitopes and provide a toolkit for toxin quantitation in vivo
Source: PLoS Pathog. 2023 Oct 23;19(10):e1011496. doi: 10.1371/journal.ppat.1011496 (PMC10621975; doi:10.1371/journal.ppat.1011496)
Supplement: S3 Table — (DOCX) [file ppat.1011496.s008.docx]

| **S3 Table. Strains used in this study** | | | |
| --- | --- | --- | --- |
| **Number** | **Strain name** | **Relevant characteristics** | **Source** |
|  | *C. difficile* |  |  |
| DBLCD6 | R20291 | Wild-type BI/NAPI/027 from Nottingham Clostridia Research Group | [1] |
| DBLCD1 | R20291 *tcdA1584s::CT tcdB1578s::CT* | DBLCD6 containing two ClosTron insertions one after nucleotide 1584 of *tcdA* harboring *ermB* (Erm^r^) and another after nucleotide 1578 of *tcdB* harboring *catP* (Cam^r^). | [2] |
| DBLCD5 | R20291-TcdBGTX | DBLCD6 containing TcdB::D286N/D288N | [3] |
| DBLCD7 | VPI 10463 | Abdominal wound isolate Toxinotype 0 | ATCC 43255 |
| DBLCD24 | M7404 | Canadian B1/NAP1/027 isolate | [4] |
| DBLCD62 | M7404 *tcdA4068s::ermB* | DBLCD24 containing targetron insertion after nucleotide 4068 of *tcdA* harboring *ermB* (Erm^r^). | [4] |
| DBLCD64 | M7404 *tcdB1587s::ermB* | DBLCD24 containing targetron insertion after nucleotide 1587 of *tcdB* harboring *ermB* (Erm^r^). | [4] |
| DBLCD66 | M7404 | DBLCD64 containing targetron insertions after nucleotide 4068 of *tcdA* harboring *ermB* (Erm^r^) and after nucleotide 1587 of *tcdB* harboring *ermB* (Erm^r^). | [4] |
|  | *tcdA4068s::ermB* |  |  |
|  | *tcdB1587s::ermB* |  |  |

**References**

1. Ng YK, Ehsaan M, Philip S, Collery MM, Janoir C, Collignon A, et al. Expanding the repertoire of gene tools for precise manipulation of the Clostridium difficile genome: allelic exchange using pyrE alleles. PLoS One. 2013;8: e56051. doi:10.1371/journal.pone.0056051

2. Kuehne SA, Minton NP. ClosTron-mediated engineering of Clostridium. Bioengineered. 2012;3: 247–254. doi:10.4161/bioe.21004

3. Christopher Peritore-Galve F, Shupe JA, Cave RJ, Childress KO, Kay Washington M, Kuehne SA, et al. Glucosyltransferase-dependent and independent effects of Clostridioides difficile toxins during infection. PLOS Pathogens. 2022;18: e1010323. doi:10.1371/JOURNAL.PPAT.1010323

4. Carter GP, Chakravorty A, Pham Nguyen TA, Mileto S, Schreiber F, Li L, et al. Defining the Roles of TcdA and TcdB in Localized Gastrointestinal Disease, Systemic Organ Damage, and the Host Response during Clostridium difficile Infections. Ballard J, Collier RJ, editors. mBio. 2015;6: e00551-15. doi:10.1128/mBio.00551-15
