# Supplementary material for: Nanobodies against C. difficile TcdA and TcdB reveal unexpected neutralizing epitopes and provide a toolkit for toxin quantitation in vivo
Source: PLoS Pathog. 2023 Oct 23;19(10):e1011496. doi: 10.1371/journal.ppat.1011496 (PMC10621975; doi:10.1371/journal.ppat.1011496)
Supplement: Attachment — Submitted filename: review_response2.0.docx [file ppat.1011496.s009.docx]

Dear Dr. Koropatkin,

Thank you for your assistance in coordinating the review of our manuscript. We are grateful for the constructive critique, the enthusiastic response, and the opportunity to further clarify the presentation of our work.

We offer here a point-by-point list of our responses to all review comments, and a description of the changes we have made in the manuscript.

**Part I – Excerpt from Summary**

Reviewer 3: The assay also enables researchers to ask questions about how individual toxin levels vary over the length of an infection and correlate these measurements with disease severity. Related to this point, the authors find that similar amounts of TcdA and TcdB toxin are detected in cecal and fecal contents of mice, suggesting that the differences observed in the pathogenicity caused by TcdA vs. TcdB are due to differences in activity/ host cell targeting rather than levels per se (some reports suggest that TcdB levels might exceed TcdA, but they show that this is not the case). *The authors may want to specifically comment on this finding in their Discussion section.*

We have expanded on this in the Discussion, lines 405-414.

In the conditions of this study, we observed significant variation in the quantities of TcdA and TcdB present within the cecal contents of mice at two days post infection (Table 6) and no correlation between toxin levels and weight loss (Supplemental Figure 4). This may suggest that differences in pathogenic responses caused by TcdA and TcdB are due to differences in activity and the host response rather than toxin levels. We note, however, that these samples were evaluated at a single time point and that weight loss is an indirect readout for mechanistic study. An important priority for future studies will be to evaluate toxin levels at earlier timepoints and to identify alternate and/or more direct readouts of toxin activity that can be used to predict disease outcomes in the host.

**Part II – Major Issues: Key Experiments Required for Acceptance**

Reviewer #1: No major concerns.

Reviewer #2:

1. Is anything known about the absolute or relative binding affinities of the different HA-tagged Nbs to the toxins? Might differences in neutralization properties reflect differences in binding affinity? Seems that this information is important particularly as the investigators are proposing that others use these nanobodies as tools in their research. Furthermore, might differences in binding affinities explain the sensitivity of the sandwich ELISAs conducted with different pairs of Nbs? As well as differences in their ability to neutralize? For example, can one conclude that the two toxins are differentially neutralized by Nbs that bind the CROPS domains of the two toxins, if it is not known whether those Nbs being compared bind to the CROPS domains with similar binding affinities?

We have not determined affinities for this panel of 42 nanobodies. Neutralization properties against the same epitopes are expected to correlate well with affinity. However, neutralization is largely determined by epitope. Some epitopes bind antibodies with high affinity, but there are no structural or functional consequences that lead to the neutralization of toxin activity. In other cases, the epitope is associated with a key function such as receptor binding or pore formation, and the consequence of even a low affinity nanobody binding event can disrupt or neutralize toxin function. In some cases, these two types of epitopes are located on the same toxin domain, as seen here with some our TcdB GTD nanobodies B0D11 and B2C11. Since we do not yet know the exact epitopes for our panel, we are not yet able to assess the impact of differences in binding affinity.

Similarly, we agree that binding affinity could be a key determinant of sandwich ELISA sensitivity when comparing nanobodies that target the same epitope. However, in addition to the fact that we don’t yet know which nanobodies bind the same epitopes, the ELISA sensitivity can also be impacted by how different capture nanobodies adhere to the plate, and the accessibility of the detection epitope once bound by the capture nanobody.

In the specific question of the mechanism by which toxins are neutralized by CROPS targeted nanobodies, the affinity is expected to be one of several important determinants. The CROPS region is known to bind glycans, but the glycan preferences differ between TcdA and TcdB. In addition, the CROPS is highly repetitive with 7 repeating units in TcdA and 4 in TcdB. In both our work and the work of others, many CROPS specific Fabs and nanobodies bind to multiple sites along the CROPS, and therefore the other experimental variable to consider is the stoichiometry of binding. In short, there are a lot of open questions about the role of glycan binding and repeating sequence elements that need to be addressed in order to fully understand the mechanisms of neutralization associated with CROPS-directed nanobodies.

Overall, we agree that the nanobody binding affinities are an important determinant of neutralization potency. The ability to compare between nanobodies however will require a detailed structural understanding of which nanobodies bind to shared epitopes and is outside the scope of this initial report. To clarify that the affinity will be one of the important variables to consider going forward, we have added a line to the discussion (lines 383-384):

There are several factors that are expected to influence the potency of neutralization: epitope, binding affinity, and stoichiometry.

2. In the data availability statement, it says that “Sequences to clones developed in this work can be found in the supplemental figures”. Presumably. this sentence is referring to the sequences of the nanobodies. However, I was unable to find this information anywhere in the supplemental materials. Given that the focus of this manuscript is the characterization of the anti-TcdA and anti-TcdB nanobodies and their potential to be used by others in future studies, a supplemental table that contains the unique sequences of all the 69 anti-TcdA and 126 anti-TcdB nanobodies isolated should be added. In such a table, it would be helpful to demarcate which of the sequences of the nanobodies map to their V, D, J and CDR3 regions.

We have included the table of sequences with CDRs indicated in Supplemental Table 1

3. Is detection of toxins in the feces and/or cecal contents improved when fecal material is filtered?

Unfortunately, no. Efforts to filter introduced variation due to the small volumes. We have clarified this point in lines 593-594: Unlike samples secreted into TY media, cecal and fecal samples could not be reliably filtered.

Reviewer #3: (No Response)

**Part III – Minor Issues: Editorial and Data Presentation Modifications**
Please use this section for editorial suggestions as well as relatively minor modifications of existing data that would enhance clarity.

Reviewer #1: Some clarifications throughout would be helpful to readers. Specifically:
(1) As was mentioned in discussion, please modify methods section to provide a clear timeline for alpaca immunizations and bleeds. A clearer rationale for the number of immunizations and their timing, and any information tracking titers would be helpful.

(2) Please provide more information on Gerbu adjuvant (formulation, composition, supplier).

Thank you for noting these omissions. We have clarified points (1) and (2) in the methods section (lines 426-430) with the following text: “Two alpacas, one for each toxin, were immunized 8 times with 125 µg of purified mutant toxin mixed 1:1 by volume in Gerbu adjuvant FAMA (item 3030 Gerbu F from GERBU Biotechnik). Animals were immunized on days 0, 14, 29, 42, 55, 69, 90, and 104. Blood was drawn on days 42, 78, and 111, and similarly strong titers were seen at all timepoints.”

(3) Please comment on what B cells are being sequenced in PBMCs from immunized alpacas. Given that 8 bleeds have taken place and that they were spaced 2-3 weeks apart, presumably there are toxin-specific activated Bcells and memory B cells, as well as plasmablasts, and plasma cells.

Total PBMC’s were harvested. Cells were not sequenced, but rather, total RNA was purified and used in a nested PCR strategy to isolate nanobody fragments. We have clarified this in lines 147-150 of the results and line 481 of the methods.

Text added in lines 146-150 for clarification: Following immunization, blood was drawn, peripheral blood mononuclear cells (PBMCs) were isolated, total RNA was purified, cDNA was made, and phage display libraries were produced. These libraries are not cell type specific, include representation from all circulating B-cells, and are not B-cell lineage specific.

Text added in line 437-438 for clarification: The resulting PCR fragments arise from B-cells of diverse lineages.

(4) A sentence of two for the benefit of non-specialists to better understand negative staining electron microscopy would be helpful.

We have added a brief description in lines 221-226: Negative stain EM is a method in which dispersed proteins or complexes are coated with a high contrast stain and imaged with an electron microscope. Many orientations are imaged simultaneously, and individual particles are then aligned and grouped by their shape and orientation. The signal enhancement obtained by averaging particles within a class or group can often provide an indication of two-dimensional shape and size.

(5) The clonality of the alpaca nanobody response to each toxin is clearly described but its relevance to human responses to those toxins is not clear. Given, the variety of B lineage types in the PBMCs, it should be acknowledged that the data do not reveal the clonality of specific lineages (memory versus plasma cell for example).

Thank you for this suggestion. We have clarified this point as part of our response to (3), above.

(6) Can the authors discuss whether the difference in nanobody recognition of different epitopes on TcdA versus TcdB extrapolate to other species? Since Ab recognition in mouse and human seem to concentrate in the CROP regions of TcdB, albeit not exclusively in that region, are similar differences observed between responses to TcdA and TcdB in these species?

We would love to have a way to address this important question. We are beginning a search for human antibodies to address this in future studies. Unfortunately, at this point we cannot add to this discussion.

Reviewer #2:

1. It would be useful for the reader to divide results section into subsections with headers.

Thank you for this suggestion. We have added headers for subsections (Nanobody Development, Toxin Neutralization, Epitope Identification, ELISA Development) within the results section.

2. Line 173: Are the number of potential V, D and J regions of heavy chain antibodies known? If so, might provide some context to provide some background information in this area.

Thank you for the opportunity to address this. The numbers of V, D, and J regions are known and annotated in IMGT. There are 17 V regions associated with VHH antibodies, 8 D regions, and 7 J regions. We have added the text “As annotated in IMGT, there are 17 V regions associated with VHH antibodies in alpacas, 8 D regions, and 7 J regions [32], highlighting the constrained gene usage and the importance of CDR3, which is often critical for antigen recognition” in lines 185-188. To our knowledge a single study has addressed variable gene usage in alpacas (PMID: 32506493) and this study has found very skewed usage with the same dominant genes we identify. This is described in the second paragraph of the discussion with the citation being reference 36.

3. Line 180: Would provide more background and clarify that the complementarity determining regions are those that bind the antigen.

Thank you for this suggestion. We have expanded this section as noted in (2). We now clarify, lines 187-188, that CDR3 is “often critical for antigen recognition.” There is also some additional description of CDRs in the end of the introduction, lines 120-124.

4. Line 186/188. Would consider adding labeling the two clades in sFig. 1 A and B as referred to in the text. In the figure, they are referred to as top and bottom panels.

Thank you, we have properly labeled the panels.

5. Lines 195, as both cell lines were not used with each nanobody, would change and to and/or. Please include information and references regarding the TcdA and TcdB receptors known to be present on Vero, Caco-2 and T84 cells. What does it mean when the EC50 is vastly different between cell types, i.e., with A1G4? Would also define EC50 somewhere in the text.

We have made the change to and/or.

We have added text to lines 203-209 to better justify our reason for screening two cell types, and a reference documenting receptor profile differences across different cell types:

Two cell lines were screened for each toxin to address potential differences in surface receptor repertoires present on the cells. In the case of TcdA where the relevant receptor repertoire remains undefined, nanobody-dependent neutralization was assessed using T84 and/or Vero cells. For TcdB, Caco-2 and/or Vero cells were used. It is known that the TcdB1 receptor, CSPG4, is present on Vero cells but not Caco2 cells and that the neutralization properties of antibodies can vary depending on cell type.

We have defined EC50 in lines 522-524: ‘For these experiments, EC50 is the concentration of nanobody that increases viability to halfway between the zero nanobody baseline and maximum achievable protection.’

6. The text in Figures S3 and S4 is not legible and unable to evaluate this data. Text in Fig. S5 is very pigmented when blown up. Were these figures really made using Biorender? Doesn’t seem like right software for this.

We apologize for this oversight and have corrected the problem so that these images should now be clear.

7. Line 253-254. I think the investigators are referring to Figure S3 not Figure S2.

Thank you for noting this. We have made the change.

8. Reference to Fig. S5 precedes Fig. S4.

We have changed the order of Fig. S5 and Fig. S4 so that they are presented sequentially in the text.

9. Line 268-269: Why focus on developing an assay to measure TcdB1 and TcdB2 as opposed to all five TcdB subtypes? Is it feasible that in future studies a standardize clinical sandwich ELISA could be developed to diagnose all five toxin subfamilies? Thoughts reflecting this possibility would be interesting to include in the discussion.

We started with TcdB1 and TcdB2 as these are the sequences found in most clinical isolates. However, we do think it is feasible to develop an assay to detect all TcdB members and hope to tackle this in our on-going work. We have added clarifying text to the results on lines 287-288.

Reviewer #3: The authors find that there is no correlation between the levels of toxins measured and the weight loss observed in individual mice. This could suggest that there are additional factors that drive disease severity beyond the toxins (e.g. host responses). Since the authors point out in their discussion that the ELISA assay does not distinguish between whether toxins are intact and functional, it would be helpful to determine if the toxin levels they measure correlate with bulk toxin activity (TcdA + TcdB combined) using the standard Vero cell assay. This information would allow them to demonstrate that their ELISA assay is a good proxy for toxin levels and activity in in vivo samples. Aside from this experiment, which is not essential for publication, I have minor suggestions on data presentation and the Discussion points.

While not a goal for this study, we agree that an investigation of how the toxin levels, as measured by ELISA, correlate with toxin activity assays, could allow us to use toxin concentration measurements as a proxy for toxin activity in vivo. While theoretically feasible, there will be some technical hurdles to work through since the strain we used for infection, R20291, produces the *C. difficile* binary toxin which also causes cell rounding.

Speaking more broadly, however, we do expect weight loss to result from a complex set of factors that include the toxins, the host, and the microbiota. An important priority for future studies will be to identify alternate and/or more direct readouts of toxin activity that can be used to predict disease outcomes in the host. We have added to the Discussion (lines 405-415) to include these points for readers:

For some strains, such as the R20291 strain used in this study, one will also need to neutralize the rounding associated with the CDT binary toxin.

In the conditions of this study, we observed significant variation in the quantities of TcdA and TcdB present within the cecal contents of mice at two days post infection (Table 6) and no correlation between toxin levels and weight loss (Supplemental Figure 4). This may suggest that differences in pathogenic responses caused by TcdA and TcdB are due to differences in activity and the host response rather than toxin levels. We note, however, that we used a strain where the glucosyltransferase activity of TcdB had been inactivated, the samples were evaluated at a single time point, and weight loss is an indirect readout for mechanistic study. An important priority for future studies will be to evaluate toxin levels at earlier timepoints and to identify alternate and/or more direct readouts of toxin activity that can be used to predict disease outcomes in the host.

Related to the earlier point, it would be helpful to move Supplemental Figure 5 to the main paper, since an important contribution of the paper is their systematic demonstration that toxin levels do not necessarily correlate with disease symptoms.

We acknowledge the rationale for doing this. On the other hand, we are always cautious about the evidence needed to draw a conclusion from a negative result. Perhaps we needed more replicates, or perhaps we should have looked 12 hours earlier? We do think it valuable to share our observations at this time point, and we hope it will motivate others to use these or similar methods to investigate the question in greater depth. We would favor leaving it in the supplement but are willing to move it if the editor and/or other reviewers agree.

Do the authors think that they can identify conserved epitopes with their approach that will broadly neutralize or identify the diverse toxin types, especially for TcdB? Their data currently suggest that the nanobodies against TcdB are not able to recognize different toxinotypes.

Yes, we do think we can identify broad neutralizers and broad binders. The current data suggest that some clones recognize both TcdB1 and TcdB2. For example, panel 4G shows essentially indistinguishable recognition for TcdBVPI (TcdB1) and TcdB027 (TcdB2). We have changed lines 292-294 to clarify this point. The altered text now reads: Some recognized TcdB1 and TcdB2 equally well and some were effective against TcdB1 (VPI10463) but lacked sensitivity for TcdB2 (TcdB027) (Supplemental Figure S4, panels C, E and F). We chose to pursue B2C11 and B0D10 as capture nanobodies, and B0E2 was biotinylated and used for detection. Both pairs were effective in recognizing TcdB1and TcdB2 sequences (Figure 4A, G).

Why did the authors pursue a different immunization strategy for TcdA vs. TcdB (different mutations were used to immunize llamas.)? It would be helpful to provide this information in the manuscript, since the authors have previously shown that TcdB is more conformationally flexible than TcdA. It would also be helpful to comment on why they observe differences in the neutralization activity of their nanobodies for TcdA vs. TcdB in terms of where the nanobodies target and how that can lead to toxin neutralization.

An important consideration was how to ensure the safety of the animal while preserving the native structure and epitope presentation of the toxins. Previous work from our lab as well as the labs of others have shown that TcdA can be inactivated through mutation of the glucosyltransferase domain. However, TcdB is known to have a glucosyltransferase-independent activity that can be cytotoxic at high concentrations. We referred to this work in the introduction (lines 130-135). We think that both toxins are conformational flexible in solution, so this was not a consideration when designing the immunization experiments.

Why did the authors use TcdB2 GTD instead of TcdB1?

TcdB2 GTD was readily available, and we wanted to identify cross-neutralizing nanobodies.

What was the rationale for choosing an antibody as a capture vs. a detection nanobody?

For TcdB this was largely done empirically. For TcdA, clone A1C3 emerged early in this study as a non-neutralizer that bound well and bound a unique site among our panel of clones. This was largely serendipitous. We have since developed strategies to directly test for capture efficiency, but this was not done for this project.

I would suggest adding a final model figure that shows where the most potent antibodies bind and how the antibodies performed in the ELISA assay to summarize their findings in a more visual manner to highlight the differences in the TcdA and TcdB nanobody inhibitory function that are summarized in the Discussion section.

While a great idea, we really only know the binding locations for the nanobodies we evaluated by negative stain EM, and even then, we only know these in two dimensions. This is something we hope to work toward in our on-going work.

The authors might want to consider revising Table 1 so that it is incorporated into part of Figure 2 as a heat map rather than a table.

Thank you for the suggestion. Unfortunately, we have not found a figure design that is clearer than the table.

Line 91: consider changing “and” to “but” We have made this change.
Line 95: consider adding “repetitive” before “sequence blocks” We have made this change.
Line 256: please add a comma before “while” We have made this change.
Line 297: please hyphenate “domain-specific” We have made this change.
Line 318: the comma after “or” can be deleted; consider revising the sentence so that it reads “… role for CROPS-dependent glycan binding or as-yet-unidentified protein receptors for TcdB.” Yes, this is much simpler. We have made this change.

Line 323: this sentence was a little hard to parse. Consider “However, in this instance, binding of PA41 to the GTD blocks pore formation or the ability of the GTD to be transited through the endosomal pore.”

We have changed the sentence to: *In that instance, PA41 binds GTD and blocks either pore formation or translocation of GTD through the endosomal pore, perhaps by blocking GTD unfolding.*

Line 328: consider replacing “In addition to autoprocessing and release of the GTD” with “This is because”

*We have changed this sentence to: This is because the toxins are subjected to exogenous proteases within the complex milieu of the intestinal tract and GTD is known to be released by autoprocessing.*

Line 332: the comma on this line can be deleted We have made this change.
Line 347: consider revising the sentence so that it reads “If it is true that binding sites are being disrupted…also consider toxin epitope availability within the complex environment of the intestinal lumen” (add “it is” and delete “of” before “environment”) We have made this change.
Line 370: “the” can be deleted We have made this change.
Line 371: consider replacing “complex” with “heterogeneous nature of these samples” We have made this change.

Line 307: please define what “variable gene usage” is

Thank you. This was unclear, so we have changed the sentence to: *Despite broad domain coverage, both the TcdA and TcdB nanobody panels were composed of a relatively small number of antibody variable genes. (lines 331 and 332)*
